# Intracardiac migration of a left renal vein stent after endovascular treatment of nutcracker-associated pelvic congestion syndrome: a case report

**DOI:** 10.1186/s12893-026-03848-6

**Published:** 2026-05-23

**Authors:** Muhammad Hamouda, Bernd Muehling

**Affiliations:** Department of Cardiac, Thoracic and Vascular Surgery, University Hospital Ulm (Universitätsklinikum Ulm), Albert-Einstein-Allee 23, Ulm, 89081 Germany

**Keywords:** Nutcracker syndrome, Pelvic congestion syndrome, Venous stent, Stent migration, Tricuspid valve, Chronic pelvic pain

## Abstract

**Background:**

Left renal vein (LRV) stenting is used in selected patients with symptomatic Nutcracker syndrome (NCS). Stent migration is uncommon but may be serious, particularly in interventions performed primarily for symptom relief. We report intracardiac migration after combined treatment of NCS-associated pelvic congestion syndrome (PCS) and use the event to discuss patient selection, treatment sequencing, and consent in pain-driven venous interventions.

**Case presentation:**

We report a 37-year-old woman with chronic pelvic pain and imaging findings compatible with nutcracker syndrome and associated pelvic congestion syndrome who underwent left renal vein stenting (self-expanding nitinol stent, 14 × 40 mm) with concomitant left ovarian vein coil embolisation. Fourteen days later, she presented with acute dyspnoea and palpitations. Echocardiography revealed a linear foreign body at the tricuspid valve level with severe tricuspid regurgitation, and computed tomography confirmed stent migration from the left renal vein into the right atrium and ventricle. Endovascular snare retrieval failed because of engagement of the stent struts within the tricuspid apparatus, and surgical extraction under cardiopulmonary bypass with tricuspid valve repair was required. Despite technical success of the venous interventions, pelvic pain did not improve durably.

**Conclusions:**

Intracardiac migration after LRV stenting is rare but carries substantial clinical and ethical implications in symptom-directed venous interventions. This case illustrates the importance of a cautious, conservative-first, multidisciplinary approach to chronic pelvic pain, emphasizing careful symptom attribution in NCS-associated PCS. It also suggests that a staged treatment strategy, with reassessment of early stent stability and clinical response before considering embolisation of pelvic collateral pathways, may be considered in selected patients. Possible technical contributors include venous undersizing and limited landing zones; the role of altered flow after collateral embolisation remains speculative.

## Background

Nutcracker syndrome (NCS) describes symptomatic compression of the left renal vein (LRV), most commonly between the aorta and the superior mesenteric artery, causing renal venous hypertension and manifestations including flank pain, hematuria, and pelvic symptoms [1].

Diagnosis requires clinico-radiologic correlation, typically integrating duplex Doppler ultrasound with cross-sectional imaging. Pelvic congestion syndrome (PCS) may coexist with NCS and can reflect collateral decompression through gonadal and pelvic venous pathways, presenting with pelvic venous reflux and parauterine varices [2]. 

A central challenge is that pelvic varices and ovarian vein dilatation are common imaging findings and may correlate poorly with the presence, severity, or chronicity of pelvic pain. In addition, chronic pelvic pain is often multifactorial, and venous imaging findings alone may not establish symptom causality. Gynaecologic guidance therefore emphasizes structured evaluation and exhaustion of conservative treatment before escalation to invasive procedures, particularly when the goal is symptom relief rather than prevention of organ-threatening pathology.

However, venous stent migration has been described into the inferior vena cava, right atrium, right ventricle, and pulmonary arteries, occasionally with valvular injury and the need for open cardiac surgery [3]. 

We report a case of right-heart migration after combined endovascular treatment of NCS-associated PCS. Beyond documenting a rare complication, the case illustrates how anatomical and technical success may fail to produce durable clinical benefit, reinforcing the need for careful symptom phenotyping, conservative-first pathways, and cautious interpretation of haemodynamic and anatomical findings in symptom-directed venous interventions.

To contextualize the present case, we performed a focused descriptive literature review. The final search was performed in January 2026. PubMed and Google Scholar were searched using combinations of the terms “nutcracker syndrome,” “left renal vein,” “left renal vein stent,” “renal vein stenting,” “stent migration,” “right atrium,” and “right ventricle.” Reference lists of relevant reviews and case reports were screened for additional studies. We included English-language reports describing stent migration after left renal vein stenting performed for nutcracker syndrome. Case reports, case series, and cohort studies were considered. Duplicate reports were excluded where identifiable. A total of 12 reports or studies were included. This was a focused descriptive review and not a systematic review; no formal risk-of-bias assessment or pooled analysis was performed. 

### Case presentation

A 37-year-old woman was evaluated for chronic lower abdominal and pelvic pain with cross-sectional imaging findings interpreted as compatible with nutcracker syndrome associated with pelvic congestion syndrome. The patient reported chronic lower abdominal pain of several years’ duration, described as non-specific and of unclear origin. A prior gynaecologic evaluation did not identify a definitive cause of symptoms, although a history of mild endometriosis (status post laparoscopy in 2020) was noted. Colonoscopic evaluation performed in July 2024 was unremarkable apart from small polyps without malignant features. Conservative management had been attempted but did not result in sustained symptom relief.

**Fig. 1:**
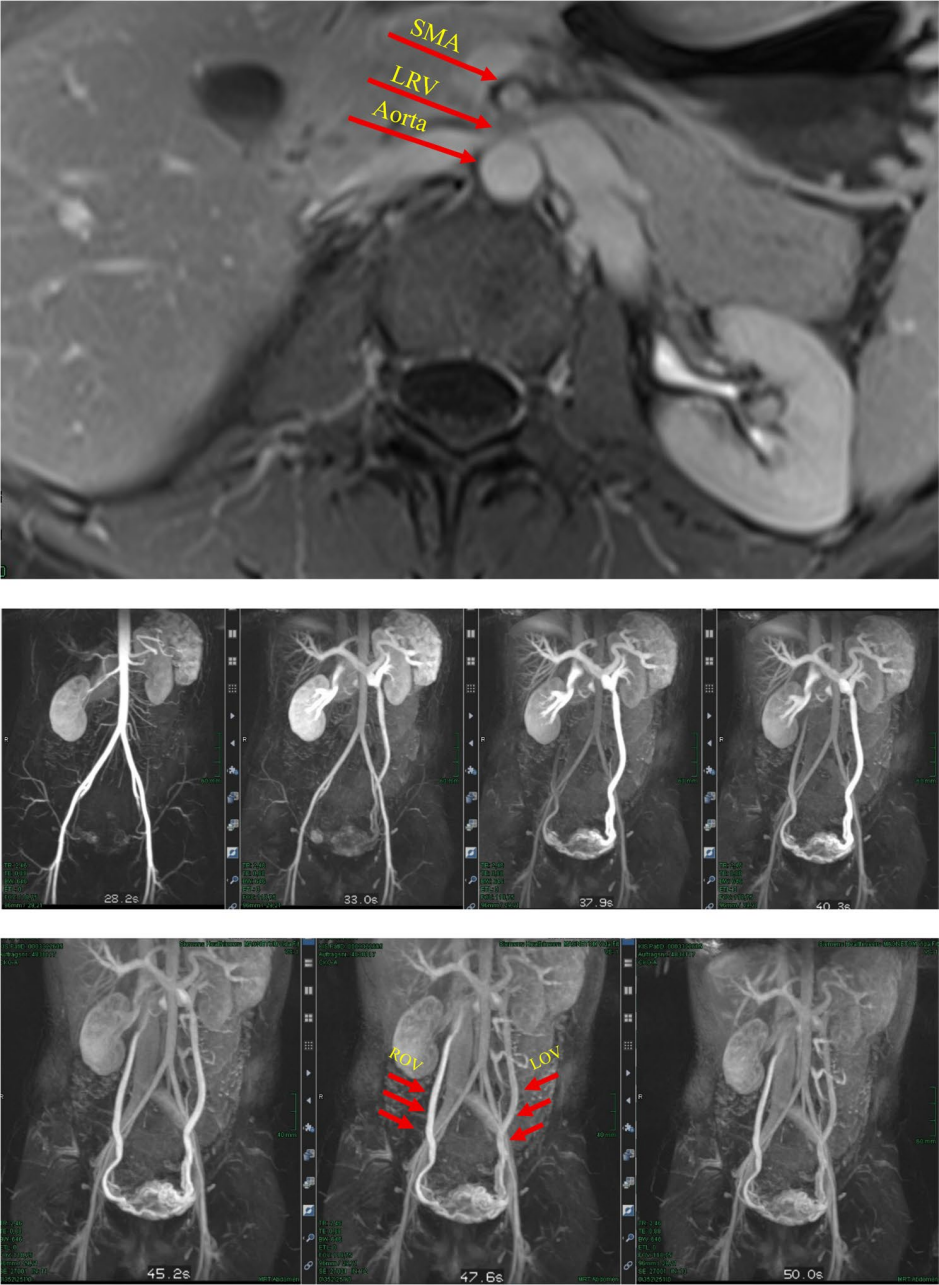
Pre-interventional imaging demonstrating high-grade compression of the left renal vein at the aorto-mesenteric crossing with collateral decompression via a markedly dilated left ovarian vein and parauterine varices, consistent with Nutcracker syndrome associated with pelvic congestion syndrome

Cross-sectional imaging (external MRI, September 2024) demonstrated a prominent parauterine venous plexus with suspected pelvic congestion syndrome. Duplex ultrasonography confirmed a dilated ovarian vein and pelvic venous plexus. Quantitative morphologic parameters of left renal vein compression, including aortomesenteric angle and distance, as well as precise measurements of the compressed and reference vein diameters, were not systematically recorded. Renocaval pressure gradients were not measured, and intravascular ultrasound (IVUS) was not used during the index procedure. Additional objective haemodynamic parameters, including duplex-derived velocity ratios, were not available. The indication for intervention was therefore based primarily on imaging findings in the context of persistent symptoms, with limited objective haemodynamic confirmation. Accordingly, diagnostic certainty of clinically significant nutcracker physiology was limited. Imaging demonstrated marked narrowing of the left renal vein at the aortomesenteric crossing with collateral decompression via a markedly dilated left ovarian vein and parauterine varices, consistent with pelvic venous reflux in the setting of renal venous outflow obstruction (Fig. 1). Following interdisciplinary discussion and written informed consent, a combined single-stage endovascular approach was undertaken to address both the LRV stenosis and the pelvic venous reflux. In retrospect, and as discussed below, these findings raise the possibility that a staged strategy may be considered, in which early stent stability and symptom response are reassessed before embolisation of pelvic collateral pathways.

The index endovascular intervention was performed on 25 October 2024 under local anaesthesia. Ultrasound-guided retrograde access was obtained via the left common femoral vein, and a 7-F introducer sheath was inserted using the Seldinger technique. After administration of 5,000 IU intravenous unfractionated heparin, the left renal vein and left ovarian vein were catheterised under fluoroscopic guidance.

Digital subtraction angiography demonstrated a filiform stenosis of the left renal vein at the aortomesenteric crossing, with retrograde flow into a markedly dilated left ovarian vein (approximately 9 mm) and a prominent parauterine venous plexus, consistent with pelvic venous reflux.

The left ovarian vein was selectively catheterised using a coaxial microcatheter system, followed by embolisation with six detachable coils (ev3 Axium and Boston Scientific Interlock systems), deployed sequentially from the distal pelvic segment toward the proximal ovarian vein. Immediate post-embolisation angiography showed persistent but reduced opacification of the ovarian vein without evidence of coil migration.

Attention was then directed to the left renal vein stenosis. Initial attempts to achieve a stable guidewire position across the lesion using multiple wire configurations were unsuccessful, necessitating exchange to a 7-F Oscor sheath positioned at the inflow of the left renal vein into the inferior vena cava. In telescoping technique, a 4-F vertebral catheter was advanced into the proximal intrarenal venous branches, and angiographic measurements were used to estimate appropriate balloon and stent dimensions.

Graded overlapping predilatation was performed using a 10 × 40 mm balloon catheter (Powerflex Pro) with inflation pressures up to approximately 8 atm. Subsequently, a self-expanding nitinol stent (E-Luminexx, 14 × 40 mm) was deployed across the stenotic segment under fluoroscopic control, with satisfactory expansion. The stent was positioned across the aortomesenteric segment toward the inferior vena cava inflow; intentional protrusion into the inferior vena cava was not specifically planned. No intravascular ultrasound (IVUS) guidance was used. Stent sizing was therefore based on angiographic estimation rather than IVUS-derived reference vein measurements. No predefined oversizing strategy was applied.

**Fig. 2:**
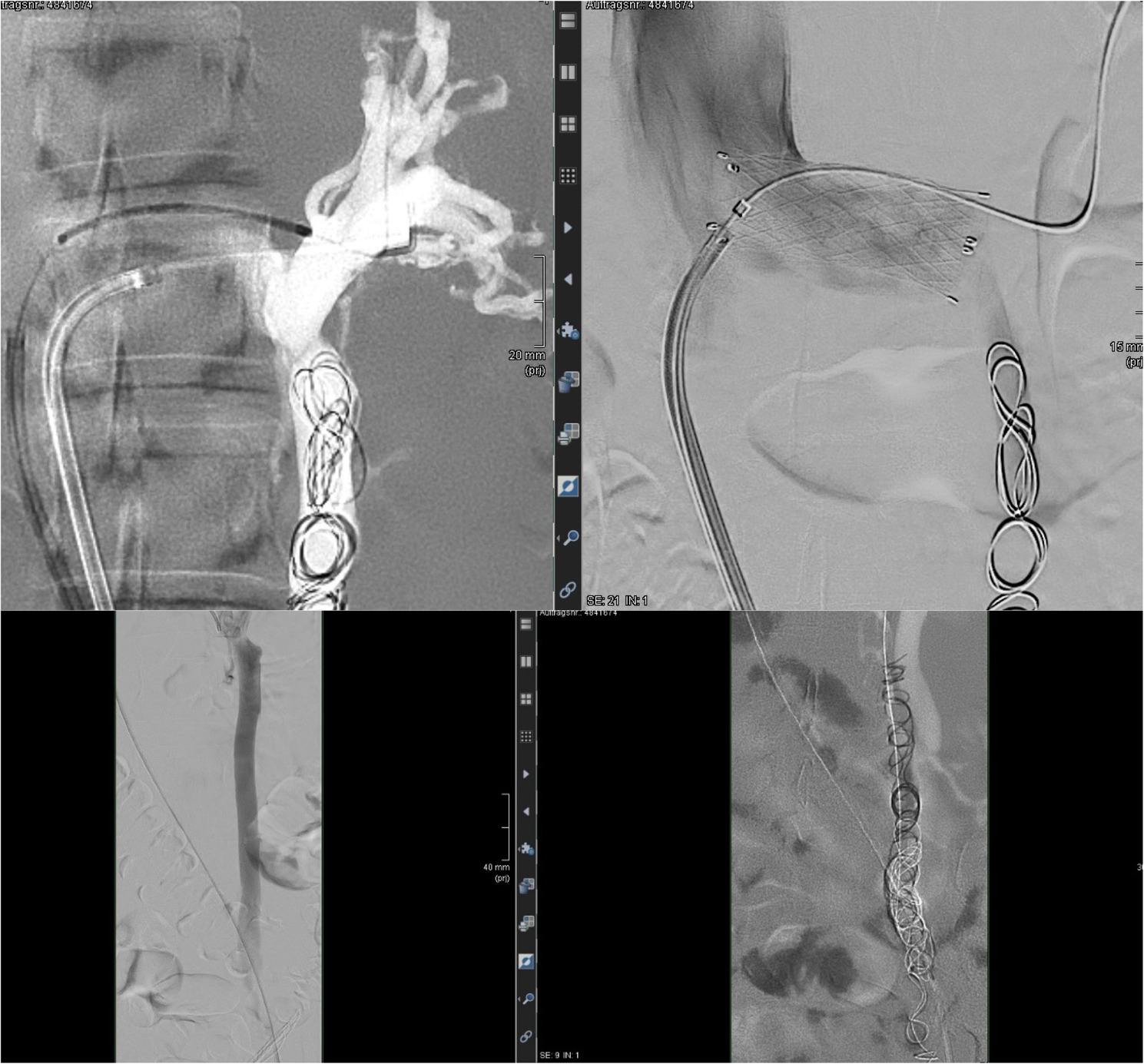
Intra-procedural venography and fluoroscopy. **A** Left renal vein venography demonstrating high-grade stenosis at the aorto-mesenteric crossing. **B** Post-stenting angiography showing restored renal venous outflow. **C** Coil embolization of the left ovarian vein performed during the same session

Completion angiography demonstrated restoration of a wide luminal calibre of the left renal vein without residual angiographic stenosis or stent waist, and markedly reduced opacification of the ovarian vein. Precise landing zone measurements and the extent of any intended protrusion into the inferior vena cava were not systematically documented. Post-dilation after stent deployment was not performed. No immediate procedural complications were observed (Fig. 2). Key patient characteristics and procedural details are summarized in Table 1.

**Table 1:** Patient characteristics and procedural details

Characteristic	Value
Age / sex	37 years / female
Body mass index (BMI)	19.3 kg/m²
Left renal vein (LRV) diameter at hilum	14 mm
Left renal vein (LRV) diameter at stenosis	not systematically documented
Ovarian vein diameter	9 mm
Stent type	E-Luminexx (nitinol)
Stent dimensions	14 × 40 mm
Time to stent migration	14 days
Tricuspid regurgitation severity	Grade 4

Post-procedural management consisted of intravenous unfractionated heparin for 48 h (target activated partial thromboplastin time 50–70 s), followed by therapeutic low-molecular-weight heparin for 14 days, and acetylsalicylic acid 100 mg daily for six months, reflecting institutional practice for venous stent implantation. Surveillance was planned with duplex ultrasonography prior to discharge and at 3–4 weeks, followed by MR angiography at three months.

**Fig. 3:**
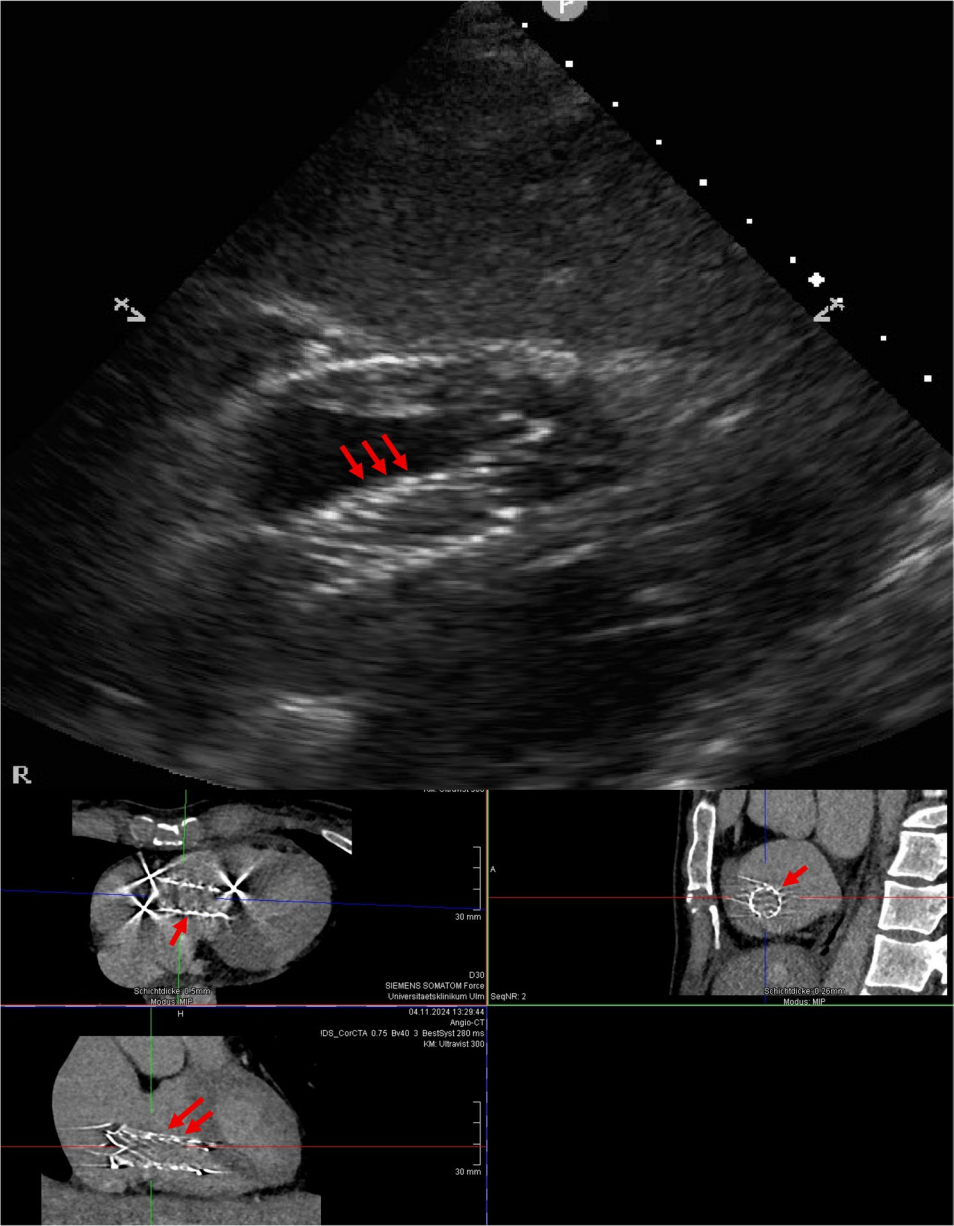
Pre-operative transthoracic echocardiography and computed tomography demonstrating a migrated left renal vein stent at the level of the tricuspid valve, extending into the right atrium and right ventricle. Arrows indicate the migrated stent. Echocardiography demonstrated associated severe tricuspid regurgitation.

Approximately two weeks later, the patient presented with acute dyspnoea and palpitations. Transthoracic echocardiography demonstrated a linear echogenic foreign body within the right heart associated with significant tricuspid regurgitation. Contrast-enhanced computed tomography confirmed migration of the LRV stent through the inferior vena cava into the right heart, with the stent positioned at the tricuspid valve level and extending into the right atrium and right ventricle (Fig. 3). 

**Fig. 4:**
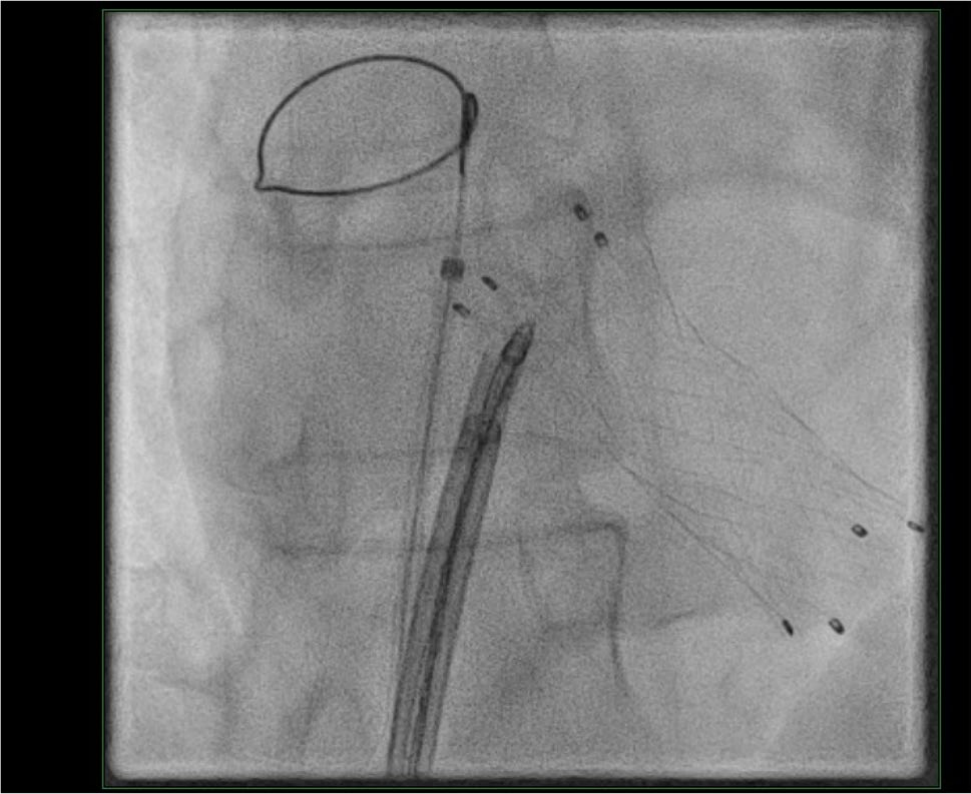
Endovascular snare retrieval attempt demonstrating engagement of the migrated stent within the tricuspid valvular apparatus

Urgent endovascular retrieval was attempted via transfemoral venous access using a 20-F sheath, a steerable introducer, and multiple snare systems under fluoroscopic and echocardiographic guidance. Retrieval was unsuccessful because of unfavourable stent orientation and mechanical entanglement of the open stent struts within the tricuspid valvular and subvalvular apparatus (Fig. 4). The stent could not be safely engaged and collapsed into the sheath. Given the risk of further valvular injury, the procedure was aborted after multidisciplinary discussion and definitive surgical management was pursued.

The patient underwent urgent surgical retrieval of the migrated stent under general anaesthesia. Transoesophageal echocardiography confirmed a metallic foreign body at the level of the tricuspid valve, causing severe tricuspid regurgitation due to impaired leaflet coaptation. Cardiopulmonary bypass was established via femoral arterial and venous cannulation, with additional venous drainage via the right internal jugular vein. After initiation of extracorporeal circulation, a minimally invasive right anterolateral thoracotomy was performed. Ventricular fibrillation was induced, the aorta was occluded using an endoaortic balloon catheter, and myocardial protection was achieved with cold crystalloid cardioplegia.

Right atriotomy revealed the migrated self-expanding nitinol stent entangled within the tricuspid valve apparatus. Multiple chordae tendineae were engaged within the open stent struts, and the valve leaflets were severely traumatised with haemorrhagic infiltration and chordal disruption. The stent was carefully dissected and completely removed without further damage to the subvalvular structures.

Intraoperative assessment demonstrated persistent severe tricuspid regurgitation due to chordal rupture involving the anterior and posterior leaflets, as well as leaflet perforation. Given the patient’s young age, valve repair was preferred over replacement. Reconstruction was performed by bicuspidalisation of the tricuspid valve and closure of the anterior leaflet perforation with 5 − 0 polypropylene sutures. Annuloplasty was not performed, as there was no significant annular dilatation.

After reperfusion, sinus rhythm was restored and the patient was weaned from cardiopulmonary bypass without difficulty. Intraoperative transoesophageal echocardiography demonstrated residual moderate tricuspid regurgitation, which was accepted to avoid valve replacement. The postoperative course was haemodynamically stable.

By the time of stent migration, the patient had not reported meaningful improvement in pelvic pain. Following cardiac surgery and stent removal, the patient reported no durable improvement in pelvic pain at 3-month follow-up.

At cardiology follow-up approximately 5–6 months after surgical stent extraction and tricuspid valve repair, the patient reported overall clinical improvement. She denied exertional dyspnoea or angina and had no current peripheral oedema. Resting sinus tachycardia persisted (heart rate approximately 90/min) with low blood pressure (112/82 mmHg). Transthoracic echocardiography demonstrated a good post-repair result with only mild residual tricuspid regurgitation and no evidence of tricuspid stenosis (mean gradient 1 mmHg). Right-sided chamber size was normal and the inferior vena cava was not dilated, with more than 50% respiratory variation. Left ventricular systolic function remained preserved (biplane ejection fraction 56%), whereas right ventricular systolic function was mildly reduced (TAPSE 16 mm). No pericardial effusion was present. A gradual return to physical activity was recommended, and planned cardiac rehabilitation was endorsed.

From a symptom perspective, chronic pelvic pain did not improve durably despite radiological correction of venous anatomy and embolisation of the pelvic reflux pathway. Given the absence of sustained pain benefit and the severity of the complication, further invasive venous escalation was deferred. The patient was referred to a structured multidisciplinary pathway for chronic pelvic pain management in line with gynaecologic and pain-medicine guidance. 

## Discussion and conclusions

As summarized in Tables 2 and 3, stent migration after left renal vein stenting appears to be uncommon and most often involves short-distance displacement into the inferior vena cava, whereas intracardiac migration has been described only in isolated reports. In symptom-directed interventions, this complication carries clinical and ethical weight because the primary objective is often pain relief rather than prevention of organ-threatening pathology [4]. 

**Table 2:** Published cohort studies and case series reporting left renal vein stent migration in Nutcracker syndrome

Study (Year)	Study design	Population	Patients / Stents (*n*)	Migrations (*n*)	Reported migration rate	Migration sites reported	Notes
Hartung et al. (2005)[5]	Case series	Adult NCS	5	≥ 3	Not precisely stated	Inferior vena cava	Early experience; Wallstents; recurrent compression/reintervention described
Chen et al. (2015) [6]	Retrospective cohort	Adult NCS	61	3	4.9%	Right atrium; inferior vena cava; renal hilum	Includes intracardiac migration reported
Wang et al. (2012) [7]	Retrospective cohort	NCS (13–32 years)	30	2	6.7%	Inferior vena cava (both at ~ 12 months)	Both stents prolapsed into the IVC; uneventful follow-up (49 and 56 months)
Wu et al. (2016) [4]	Retrospective cohort	Adult NCS	75	5	6.7%	Inferior vena cava; right atrium; right ventricle; left renal vein	Largest cohort; intracardiac migration reported
Avgerinos et al. (2019) [8]	Multicenter cohort	Adult NCS	18	0	0%	None	No migration on mid-term follow-up
Li et al. (2013) [9]	Case report	NCS (7–31 years)	6	0	0%	None	Small series; no migration observed

Migration rates are study-specific and reflect heterogeneous populations, techniques, and follow-up durations. Data are presented descriptively and do not allow pooled incidence estimation

**Table 3:** Reported cases of intracardiac migration of left renal vein stents

Author (Year)	Age / Sex	Indication	Time to migration	Stent location	Retrieval method	Cardiac injury / outcome
Chen et al. (2009) [10]	27 / M	Nutcracker	Early	Right atrium	Open cardiac surgery	Recovered; no sequelae
Chen et al. (2015) [11]	32 / M	Nutcracker	5 months	Right ventricle	Open surgery + valve replacement	Severe TR; prosthetic valve
Sebastian et al. (2017)[12]	39 / F	Nutcracker	2 days	Right ventricle	Endovascular retrieval	No valve injury
Zhang & Li (2022) [13]	41 / M	Nutcracker	1 month	Right ventricle	Open cardiac surgery	Tricuspid valve involvement
Luo et al. (2023) [14]	62 / F	Nutcracker	5 days	Right ventricle	Endovascular retrieval	No valve injury
Cooley et al. (2023) [15]	30 / F	Nutcracker + PCS	6 months	Pulmonary artery (via RA/RV)	Retrieval failed; left in situ	Managed conservatively
Present case	37 / F	Nutcracker + PCS	2 weeks	Right atrium (tricuspid valve level with RA/RV extension)	Open cardiac surgery + stent extraction	Tricuspid valve repair; residual TR accepted intraoperatively

Published cohort studies and small case series report generally favourable technical outcomes after left renal vein (LRV) stenting in selected patients with Nutcracker syndrome (NCS), although migration rates vary and intracardiac events have been described [4, 5, 7–9]. 

However, migration is a recognized complication with uniquely serious consequences because the venous outflow tract leads directly to the right heart. Intracardiac migration into the right atrium or right ventricle has been reported and may be associated with tricuspid valve injury, arrhythmia, and the need for open cardiac surgery. Even when rare, these outcomes are clinically consequential in young patients undergoing preference-sensitive procedures primarily for pain control [4, 11, 16]. 

When feasible, published reports typically describe an initial attempt at endovascular retrieval. In practice, intracardiac stents may be unfavourably oriented, partially embedded, or entangled within valvular or subvalvular structures, making endovascular extraction unsuccessful or unsafe and necessitating surgical removal under cardiopulmonary bypass. The course in the present patient reflects this pattern, and the need for tricuspid valve repair highlights the potential for secondary cardiac injury associated with venous stent migration [12, 17]. 

A further lesson from this case concerns procedural sequencing in NCS-associated pelvic congestion syndrome (PCS). In this setting, pelvic venous reflux may represent collateral decompression—an “escape” pathway—for renal venous hypertension. Occluding ovarian or pelvic veins may alter venous flow distribution along the renal vein–caval axis; however, in the absence of direct haemodynamic assessment, any contribution of this effect to migration in the present case remains speculative. Migration has been reported after combined or closely staged LRV stenting and ovarian vein embolisation [18]. 

Although causality cannot be inferred from case reports, this recurring association raises the hypothesis that a cautious staged strategy may be considered in selected patients, in which the primary renal venous outflow lesion is addressed first, followed by reassessment of early stent stability and symptom phenotype before considering occlusion of pelvic collateral pathways—particularly when the primary indication is pain relief.

Venous compression syndromes such as NCS, and their association with PCS, are best approached as clinico-radiologic entities rather than being assumed to represent the sole explanation for chronic pelvic pain. While renal venous hypertension with collateral decompression via gonadal and pelvic veins provides a plausible haemodynamic mechanism, imaging abnormalities alone cannot establish causality for chronic pelvic pain. Pelvic varices and ovarian vein dilatation are frequently seen on CT and MRI, including in asymptomatic women, and correlations with pain severity are inconsistent. This creates an interpretive environment in which striking anatomy may narrow the differential diagnosis prematurely and encourage escalation to invasive procedures whose principal aim is symptom relief rather than prevention of organ injury [19–25]. 

In the present case, the initial intervention achieved anatomical success but did not translate into durable pain relief. This is compatible with the broader chronic pelvic pain literature, in which symptoms may reflect overlapping visceral, myofascial, neuropathic, and central sensitization mechanisms. The presence of known endometriosis and the absence of objective haemodynamic confirmation further illustrate the complexity of symptom attribution in this case [25, 26]. 

Such mechanisms may coexist with venous abnormalities or dominate the clinical picture even when venous findings appear compelling. The lack of sustained analgesic benefit therefore supports a conservative-first posture and maintenance of diagnostic breadth despite dramatic venous imaging findings [27–29]. 

Technical factors may also have influenced migration risk in this compliant venous system. Possible contributors include undersizing, inadequate wall apposition, and insufficient landing zones, particularly when sizing is based on angiography alone rather than true reference dimensions of the non-stenotic vein. In the present case, the absence of intravascular ultrasound (IVUS) limited objective sizing and landing-zone assessment. Although NCS-specific comparative data are limited, IVUS is conceptually attractive when anatomy is complex and the consequences of sizing error are potentially severe [30–33]. 

Emerging technologies such as Internet-of-Things–based systems may support vascular care through remote monitoring, integrated perioperative data platforms, and structured follow-up. Although not directly relevant to the present case, such approaches may facilitate earlier detection of complications after endovascular procedures [34]. 

Three-dimensional printing is increasingly used in vascular surgery for anatomical modelling, procedural planning, and simulation. In venous compression syndromes, patient-specific models may help illustrate complex anatomical relationships between the aorta, superior mesenteric artery, left renal vein, and collateral venous pathways, although their routine role in nutcracker syndrome remains limited [35]. 

Because the indication for intervention is commonly symptom control, meticulous informed consent is central. Shared decision-making should explicitly address uncertainty regarding pain causality, variability of symptomatic response, conservative alternatives, and the permanence of implanted devices, while also explaining worst-case complications even when rare. In this context, diagnostic stewardship and conservative-first pathways may help reduce disproportionate harm when anatomical abnormalities are treated for pain phenotypes that may not be primarily venous in origin [36]. 

Intracardiac migration of a left renal vein stent after endovascular treatment of nutcracker syndrome is exceptionally rare but potentially severe. In this patient, endovascular retrieval was unsuccessful and definitive management required open surgical extraction under cardiopulmonary bypass with concomitant tricuspid valve repair, illustrating the severity of harm that may result from migration into the right heart. Equally important, the intervention achieved anatomical success without durable symptomatic benefit, underscoring the imperfect relationship between venous imaging findings and chronic pelvic pain. A pathophysiology- and pain-phenotype–informed approach that prioritizes careful symptom attribution, multidisciplinary conservative management, meticulous informed consent, and cautious staging of venous intervention may help mitigate overtreatment and serious harm in selected patients undergoing symptom-directed procedures [15, 16]. 

This single-case report has several limitations. The rarity of intracardiac stent migration after left renal vein stenting precludes identification of specific causative factors or establishment of definitive causal relationships between procedural variables and migration risk. Baseline pain was not systematically documented using validated patient-reported outcome measures, limiting quantitative evaluation of symptomatic response. Objective haemodynamic confirmation of clinically significant nutcracker physiology was limited, because renocaval pressure gradients were not measured, duplex-derived velocity ratios were unavailable, and IVUS was not used. Quantitative morphologic parameters of compression were also not systematically recorded. These limitations constrain both interpretation of treatment selection and mechanistic inference. Diagnostic certainty of clinically significant nutcracker physiology was therefore limited.

## Data Availability

All data generated or analysed during this study are included in this published article.

## Abbreviations

ACOG: American College of Obstetricians and Gynecologists
BMI: Body mass index
CT: Computed tomography
EAU: European Association of Urology
IVC: Inferior vena cava
IVUS: Intravascular ultrasound
LRV: Left renal vein
MR: Magnetic resonance
MRI: Magnetic resonance imaging
NCS: Nutcracker syndrome
PCS: Pelvic congestion syndrome
RA: Right atrium
RV: Right ventricle
TR: Tricuspid regurgitation
LOV: Left ovarian vein
ROV: Right ovarian vein
